# Education, substance use, and HIV risk among orphaned adolescents in Eastern Zimbabwe

**DOI:** 10.1080/17450128.2017.1332398

**Published:** 2017-10-02

**Authors:** E. L. Pufall, J. W. Eaton, L. Robertson, P. Mushati, C. Nyamukapa, S. Gregson

**Affiliations:** ^a^ Department of Infectious Disease Epidemiology, Imperial College London, London, UK; ^b^ Biomedical Research and Training Institute, Harare, Zimbabwe

**Keywords:** Orphans, alcohol, smoking, drugs, HIV, Zimbabwe

## Abstract

There is a growing interest in education as a means to reduce HIV infection in vulnerable children in sub-Saharan Africa; however, the mechanisms by which education reduces HIV infection remain uncertain. Substance use has been associated with high-risk sexual behaviour and could lie on the causal pathway between education and HIV risk. Therefore, we used multivariable regression to measure associations between: (i) orphanhood and substance use (alcohol, recreational drugs, and smoking), (ii) substance use and sexual risk behaviours, and (iii) school enrolment and substance use, in adolescents aged 15–19 years, in Eastern Zimbabwe. We found substance use to be low overall (6.4%, 3.2%, and 0.9% of males reported alcohol, drug, and cigarette use; <1% of females reported any substance use), but was more common in male maternal and double orphans than non-orphans. Substance use was positively associated with early sexual debut, number of sexual partners, and engaging in transactional sex, while school enrolment was associated with lower substance use in males. We conclude that education may reduce sexual risk behaviours and HIV infection rates among male adolescents in sub-Saharan Africa, in part, by reducing substance abuse.

## Introduction

The HIV epidemic in sub-Saharan Africa has increased the numbers of orphans and other children made vulnerable by HIV-related illness, stigma, and death within their families and communities (Zimbabwe Ministry of Health and Child Welfare, 2012). Orphans and children made vulnerable by HIV (OVC) are at an increased risk of exploitation (Stein, 2003; Subbarao & Coury, 2004), abuse (Stein, 2003; Subbarao & Coury, 2004), psychosocial distress (Atwine, Cantor-Graae, & Bajunirwe, 2005), poverty (Salaam, 2005), lower school attendance (Pufall et al., 2014; United Nations Children’s Fund [UNICEF], 2006), and acquiring sexually transmitted diseases (Birdthistle et al., 2008). Research from Manicaland Province, Zimbabwe supports these associations, with OVC is more likely to have HIV infection, early sexual activity, teenage pregnancy, and secondary school dropout than non-orphaned, non-vulnerable children (Gregson et al., 2005). These associations remained after adjusting for socio-economic status (SES), suggesting the effects are not explained by economic vulnerability alone. Prevalence of orphanhood remains high in Manicaland, with 33.2% of children aged 2–17 having lost one or both of their parents (Pufall et al., 2014). Attention has turned to understanding the causal pathways through which these vulnerabilities relate to HIV status, and to evaluating possible interventions to support OVC within their families and communities, as a part of a comprehensive response to HIV epidemics.

Although there is a protective effect of education on HIV risk (Hargreaves et al., 2008; Jukes, Simmons, & Bundy, 2008; Pettifor et al., 2008; Pufall, Nyamukapa, Eaton, Campbell, et al.,  2014), the exact mechanisms by which schooling decreases HIV risk are unclear. School education may decrease risk directly through delayed sexual debut, and by decreasing other sexual risk behaviours (e.g. entering into relationships with older partners, higher numbers of non-regular partners, and engaging in transactional sex). However, substance use has also been shown to be associated with increased sexual risk behaviours (Lopman et al., 2007; Meghdadpour, Curtis, Pettifor, & MacPhail, 2012; Nyamukapa et al., 2010; Peltzer, 2010; UNICEF, 2005) and may be an important mediating factor that has previously been neglected.

Despite the link between substance use and HIV risk behaviours, only a small number of epidemiological studies have looked at alcohol and drug use among adolescents in sub-Saharan Africa (Doku, Koivusilta, & Rimpelä, 2012; Famuyiwa, Aina, & Bankole-Oki, 2011; Mashita, Themane, Monyeki, & Kemper, 2011). Moreover, there are no published findings from Zimbabwe on this topic and further work to investigate linkages between education, substance use, and sexual risk behaviours is required. The aims of this study are (1) to describe levels and patterns of substance abuse in adolescents in Eastern Zimbabwe; (2) to test the hypothesis that substance abuse lies on the causal pathway between parental loss and increased HIV risk behaviour for orphaned adolescents by: (i) investigating whether orphaned adolescents have higher levels of substance abuse, and (ii) whether those practicing substance abuse have greater HIV risk behaviour; and (3) to test the hypothesis that substance abuse is reduced in orphaned adolescents who are enroled in school.

## Materials and methods

### Study population and data collection

The Manicaland HIV/STD Prevention Project (Manicaland study) is a population-based, open-cohort study in the Manicaland Province of Eastern Zimbabwe (Gregson et al., 2002, 2006; Lopman et al., 2008). Five rounds of the survey have been conducted since 1998, each taking approximately 2 years to complete. Each round involves a census of all households in 12 study sites (2 small towns; 2 roadside settlements; 4 subsistence farming areas; and 4 large-scale agricultural estates), followed by random sampling of individual household members aged 15–54 for interview and collection of dried blood spot samples for HIV testing, with roughly 10000 individuals interviewed in each round. Participants are asked about demographic and lifestyle factors, psychological and physical health, sexual relationships, and their knowledge about HIV. This study uses data collected from young adults aged 15–19 years from 2009–2011 in the fifth round of the Manicaland study.

Ethical approval for the Manicaland study was obtained from the Imperial College London Research Ethics Committee, the Institutional Review Board of the Biomedical Research and Training Institute, Zimbabwe, and the Medical Research Council of Zimbabwe. Written informed consent was obtained prior to survey participation, and participants were informed that, at any point, they could refuse to answer a question or decline to continue the interview.

### Exposure measures

School enrolment was measured through self-report in adolescents of schoolgoing age (taken as ages 15–18 years). Orphans were divided into maternal, paternal, and double orphans.

### Outcome measures

We collected data on whether adolescents smoke cigarettes, whether they use drugs for pleasure, and whether they drink alcohol. A summary variable to assess any form of substance use was created from the responses to these three questions. Sexual behaviour outcomes were ever having had sex, early sexual debut (defined as having had sex before age 15), number of non-regular partners in the last 30 days, engaging in transactional sex, and using condoms at the last sexual encounter.

### Data analysis

Logistic regression adjusting for age only was used to determine associations between demographic factors including parental loss, and the various forms of substance use. Demographic factors that were significant at *p* < 0.2 were included in multivariable regressions. Multivariable linear and logistic regression models adjusting for age, SES (measured using a previously described index (Lopman et al., 2007)), religion, and community type (town, estate, roadside settlement, or subsistence farming) were used to determine if orphanhood was associated with higher levels of substance use. Similar models were used to assess whether substance use, orphanhood (before and after adjusting for substance use), and education (before and after adjusting for substance use) were associated with higher levels of sexual risk behaviours. Finally, we assessed whether, among orphaned adolescents, school enrolment was associated with lower levels of substance use. Variables significant at *p* < 0.2 in age-adjusted models were included in the fully adjusted multivariable regression models. All analyses were stratified by gender.

## Results

A total of 3274 young adults aged 15–19 were included in this study. Fifty-one per cent of the study population was female and demographic characteristics were generally evenly distributed between the genders (Table S1). Overall levels of any substance use were relatively low (3.8%, 123/3273) and were higher in males (6.7%, 108/1609) than in females (0.9%, 15/1664). Males were more likely than females to report using cigarettes (0.9% vs. 0.1%), drugs (3.2% vs. 0.4%), and alcohol (6.4% vs. 0.7%) (all *p* < 0.01). Among males, smoking (0.9%, 15/1609) was reported significantly less than drug use (3.2%, 52/1606) and drinking alcohol (6.4%, 102/1604) (both *p* < 0.01), while alcohol consumption was significantly more prevalent than drug use (*p* < 0.01). Females were more likely than males to report early sexual debut (2.8% vs. 0.5%) and being sexually active (24.4% vs. 6.4%) but among those who were sexually active, females were less likely to have engaged in transactional sex (2.5% vs. 4.9%) and to have used condoms at their last sexual encounter (9.7% vs. 70.9%) (all *p* < 0.01).

**Figure 1.**
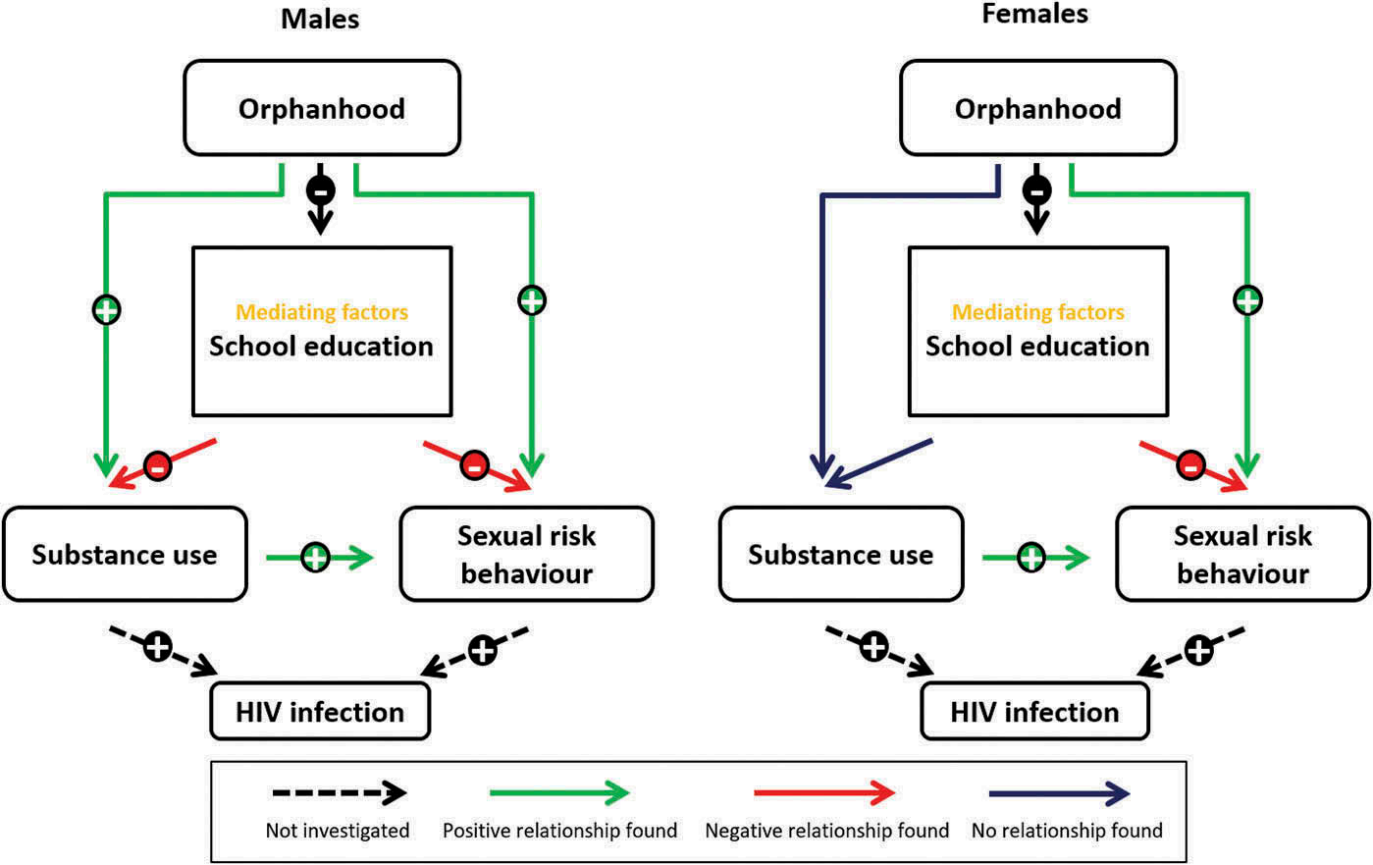
Conceptual model and observed relationships between orphanhood, substance use, education, and HIV risk behaviour.

The conceptual model for this study coded to represent a summary of the associations that were detected is presented in Figure 1. Age-adjusted associations between various socio-demographic factors, including orphanhood, and substance use are presented in Table 1. For females, overall levels of substance use were very low (<1%, Table S1) and there were insufficient cases to investigate differences in substance use by orphan or education status or associations with HIV-related sexual risk behaviours. For males, maternal (4.9%, 18/364) and double orphans (5.4%, 15/280) were more likely to take drugs for pleasure than non-orphans (2.8%, 28/781), before (Table 1) and after (Table 2) adjusting for socio-demographic factors. Alcohol consumption and smoking were also somewhat elevated in maternal and double orphans but the differences were not statistically significant. Substance use was higher in agricultural areas than in towns, and all forms were less common in Spiritual churches.

**Table 1.** Age-adjusted odds-ratios for demographic factors and different forms of substance abuse.

	Males										Females	
		Smoking cigarettes		Taking drugs for pleasure		Drinking alcohol		Any substance use			Any substance use	
	N	%	AOR (95% CI)	%	AOR (95% CI)	%	AOR (95% CI)	%	AOR (95% CI)	N	%	AOR (95% CI)
Orphanhood												
Non-orphan	789	0.9%	1	2.8%	1	6.6%	1	6.6%	1	818	1.0%	1
Maternal	364	1.4%	1.7 (0.56–4.9)	4.9%	1.9 (1.04–3.5)*	7.8%	1.3 (0.82–2.1)	8.5%	1.4 (0.88–2.2)	365	0.5%	0.54 (0.12–2.4)
Paternal	699	0.9%	0.77 (0.27–2.2)	3.6%	1.2 (0.66–2.1)	5.9%	0.80 (0.52–1.2)	6.6%	0.89 (0.59–1.3)	699	0.9%	0.83 (0.29–2.4)
Double	280	1.1%	1.1 (0.30–3.9)	5.4%	2.0 (1.04–3.7)*	7.6%	1.2 (0.72–2.0)	8.6%	1.3 (0.81–2.2)	259	0.4%	0.36 (0.05–2.8)
Community type												
Town	245	0.8%	1	0.4%	1	3.7%	1	3.7%	1	262	1.1%	1
Commercial estate	336	11.9%	1.5 (0.28–8.5)	2.7%	7.2 (0.89–57.6)	7.1%	2.2 (0.97–4.8)	7.7%	2.4 (1.1–5.3)*	347	0%	N/A
Subsistence farming	662	1.1%	1.4 (0.28–6.6)	3.8%	10.5 (1.4–78.6)*	7.1%	2.1 (1.01–4.5)*	7.4%	2.2 (1.1–4.7)*	688	1.3%	1.2 (0.32–4.4)
Roadside trading centre	366	0.5%	0.61 (0.09–4.4)	4.4%	10.5 (1.4–80.5)*	6.0%	1.6 (0.69–3.5)	6.6%	1.7 (0.78–3.9)	368	0.8%	0.74 (0.15–3.7)
SES												
Poorest quintile	293	1.4%	1	2.0%	1	4.8%	1	4.8%	1	283	0%	N/A
Second quintile	361	0.8%	0.56 (0.12–2.5)	4.2%	2.0 (0.73–5.2)	6.4%	1.3 (0.64–2.6)	7.5%	1.5 (0.78–3.1)	374	0.3%	1
Middle quintile	329	0.9%	0.48 (0.10–2.2)	4.0%	1.4 (0.50–3.7)	7.6%	1.2 (0.60–2.4)	7.9%	1.3 (0.63–2.5)	353	1.4%	5.3 (0.62–45.9)
Fourth quintile	338	0.3%	0.17 (0.02–1.5)	3.0%	1.1 (0.39–3.1)	6.0%	1.0 (0.49–2.0)	6.2%	1.0 (0.51–2.1)	311	1.0%	3.6 (0.38–35.1)
Least poor quintile	286	1.3%	0.75 (0.18–3.1)	2.4%	0.83 (0.27–2.5)	7.0%	1.1 (0.55–2.3)	7.0%	1.1 (0.54–2.3)	342	1.8%	6.6 (0.80–55.5)
Religion												
Christian	869	1.0%	1	3.7%	1	7.4%	1	7.5%	1	900	1.4%	1
Traditional	4	0%	N/A	0%	N/A	0%	N/A	0%	N/A	3	0%	N/A
Spiritual	411	0.5%	0.48 (0.10–2.3)	1.2%	0.33 (0.1–0.86)*	4.1%	0.55 (0.3–0.96)*	4.1%	0.54 (0.3–0.95)*	494	0.2%	0.14 (0.02–1.1)
Other	231	0.9%	0.87 (0.19–4.1)	3.0%	0.86 (0.37–2.0)	5.7%	0.78 (0.41–1.5)	6.5%	0.89 (0.49–1.6)	244	0.4%	0.27 (0.04–2.1)
None	89	2.2%	2.0 (0.41–9.4)	7.9%	2.0 (0.83–4.9)	9.1%	1.1 (0.50–2.5)	12.4%	1.6 (0.79–3.3)	17	0%	N/A
School enrolment^a^												
Not enroled	320	1.9%	1	5.3%	1	9.7%	1	10.6%	1	493	1.4%	1
Enroled	1055	0.3%	0.24 (0.05–1.1)	0.9%	0.27 (0.1–0.64)*	2.6%	0.37 (0.2–0.7)**	2.7%	0.34 (0.2–0.6)**	934	0.6%	0.68 (0.19–2.4)

^a^Ages 15–18; N/A: No observations in comparison group; *Significant at *p* < 0.05; **Significant at p<0.01.

**Table 2.** Effect of vulnerability and proposed protective effects on drug use, smoking, and drinking alcohol in male youth from Manicaland.

	Males								
		Smoking cigarettes		Taking drugs for pleasure		Drinking alcohol		Any substance use	
	N	%	AOR (95% CI)^b^	%	AOR (95% CI)^b^	%	AOR (95% CI)^b^	%	AOR (95% CI)^b^
Orphanhood									
Non-orphan	789	0.9%	1	2.8%	1	6.6%	1	6.6%	1
Maternal orphan	364	1.4%	1.7 (0.56–4.9)	4.9%	1.9 (1.01–3.4)*	7.8%	1.2 (0.77–2.0)	8.5%	1.3 (0.84–2.1)
Paternal orphan	699	0.9%	0.77 (0.27–2.2)	3.6%	1.2 (0.64–2.1)	5.9%	0.77 (0.50–1.2)	6.6%	0.86 (0.57–1.3)
Double orphan	280	1.1%	1.1 (0.30–3.9)	5.4%	2.0 (1.04–3.8)*	7.6%	1.1 (0.67–1.9)	8.6%	1.2 (0.76–2.0)
School enrolment – all children^a^									
Not enroled	320	1.9%	1	5.3%	1	9.7%	1	10.6%	1
Enroled	1055	0.3%	0.25 (0.06–1.1)	0.9%	0.26 (0.11–0.63)**	2.6%	0.32 (0.18–0.57)***	2.7%	0.30 (0.17–0.53)***
School enrolment – maternal orphans^a^									
Not enroled	89	2.2%	1	10.1%	1	11.5%	1	13.5%	1
Enroled	226	0%	N/A	0.9%	0.13 (0.02–0.72)*	3.1%	0.30 (0.09–0.97)*	3.1%	0.30 (0.10–0.91)*
School enrolment – paternal orphans^a^									
Not enroled	161	2.5%	1	8.1%	1	9.4%	1	11.2%	1
Enroled	431	0%	N/A	0.7%	0.15 (0.04–0.60)**	2.3%	0.36 (0.15–0.90)*	2.3%	0.32 (0.13–0.78)*
School enrolment – double orphans^a^									
Not enroled	71	1.4%	1	11.3%	1	11.4%	1	14.1%	1
Enroled	169	0%	N/A	1.2%	0.17 (0.03–0.95)*	3.6%	0.33 (0.09–1.2)	3.6%	0.32 (0.09–1.1)

^a^Ages 15–18; N/A: No observations in comparison group;

^b^Adjusted for age, site type, SES, and religion.

*, **, *** Significant at p<0.05, p<0.01, p<0.001

When testing the hypothesis that male adolescents practicing substance use have greater HIV risk behaviours, we found consistent positive associations between substance use and ever having had sex, early sexual debut, number of non-regular partners, and engaging in transactional sex (Table 3). No form of substance use had a significant association with reporting condom use throughout the last sexual encounter. We also directly examined the association between orphanhood and sexual risk behaviours and found that, although orphanhood had no associations with likelihood of early sexual debut, engaging in transactional sex, or condom use, each form of orphanhood was associated with significantly higher numbers of non-regular partners in the last 30 days (Table 3). This effect ceased to be statistically significant after adjusting for substance use.

**Table 3.** Association between orphanhood, drug use, smoking, and drinking alcohol on sexual risk behaviours in male youth from Manicaland.

	Males									
		Condom use at last sexual encounter^a^		Partners in last 30 days^a^	Engaging in transactional sex^a^		Sexual debut before the age of 15		Ever had sex	
	N	%	AOR (95% CI)^c^	Change (95% CI)^c^	%	AOR (95% CI)^c^	%	AOR (95%CI)^c^	%	AOR (95%CI)^c^
Smoking										
Non-smoker	1594	69.9%	1	0	3.2%	1	0.4%	1	5.9%	1
Smoker	15	80.0%	1.6 (0.24–10.4)	1.2 (−1.2–+3.7)	20.0%	7.6 (1.1–52.3)*	6.7%	10.0 (1.1–92.2)*	66.7%	23.6 (7.0–79.3)***
Drug use										
Does not use drugs	1555	68.4%	1	0	1.4%	1	0.4%	1	5.1%	1
Uses drugs	51	79.2%	1.4 (0.39–4.7)	0.67 (−1.1–+2.4)	16.7%	18.6 (1.8–197)*	3.9%	5.1 (0.85–30.2)	47.1%	8.0 (4.2–15.3)***
Alcohol										
Does not drink	1502	64.5%	1	0	1.6%	1	0.2%	1	4.1%	1
Drinks	102	80.5%	2.9 (0.87–9.6)	1.6 (0.01–3.1)*	9.8%	6.6 (0.58–73.9)	4.9%	14.4 (3.4–87.8)**	40.2%	8.7 (5.2–14.5)***
Any form of substance use										
No substance use	1501	64.5%	1	0	1.6%	1	0.2%	1	4.1%	1
Substance use	108	80.5%	2.9 (0.87–9.6)	1.6 (0.01–3.1)*	9.8%	6.6 (0.58–73.9)	4.6%	15.8 (3.0–83.2)**	38.0%	8.0 (4.8–13.4)***
Orphanhood										
Non-orphan	789	71.1%	1	0	2.2%	1	0.5%	1	5.7%	1
Maternal orphan	364	73.1%	0.81 (0.25–2.6)	1.4 (0.50–4.4)*	11.5%	5.4 (0.69–42.3)	0.5%	1.0 (0.21–5.2)	7.1%	1.1 (0.70–1.9)
Paternal orphan	699	67.3%	0.73 (0.27–2.0)	1.9 (0.01–3.7)*	5.8%	1.0 (0.15–6.8)	0.5%	1.1 (0.26–4.3)	7.4%	1.3 (0.82–2.0)
Double orphan	280	68.1%	0.50 (0.14–1.8)	2.5 (0.51–4.6)*	9.1%	2.3 (0.26–20.6)	0.7%	1.2 (0.23–6.1)	7.9%	1.3 (0.76–2.2)
Orphanhood – adjusting for substance use										
Non-orphan	789	71.1%	1	0	2.2%	1	0.5%	1	5.7%	1
Maternal orphan	364	73.1%	0.67 (0.19–2.3)	1.3 (−0.37–+3.0)	11.5%	4.3 (0.54–34.0)	0.5%	0.95 (0.18–5.1)	7.1%	1.0 (0.61–1.7)
Paternal orphan	699	67.3%	0.85 (0.31–2.4)	−0.1 (−1.5–+1.4)	5.8%	1.2 (0.16–8.5)	0.5%	1.2 (0.29–5.0)	7.4%	1.4 (0.87–2.2)
Double orphan	280	68.1%	0.45 (0.12–1.7)	1.7 (−0.08–+3.5)	9.1%	1.7 (0.2–14.7)	0.7%	1.3 (0.25–7.4)	7.9%	1.3 (0.72–2.2)
School enrolment^b^										
Not enroled	320	82.8%	1	0	2.9%	1	0.6%	1	11.0%	1
Enroled	1055	61.9%	0.30 (0.06–1.6)	−1.2 (−3.3–+0.9)	4.8%	1.1 (0.05–22.0)	0.3%	0.85 (0.13–5.7)	2.0%	0.35 (0.19–0.64)***
School enrolment – adjusting for substance use^b^										
Not enroled	320	82.8%	1	0	2.9%	1	0.6%	1	11.0%	1
Enroled	1055	61.9%	0.31 (0.06–1.6)	−1.1 (−3.2–+0.9)	4.8%	1.3 (0.05–32.2)	0.3%	2.0 (0.23–16.6)	2.0%	0.45 (0.24–0.86)*

^a^Among sexually active; ^b^Ages 15–18; ^c^Adjusted for age, community type, SES, and religion.

**Figure 2.**
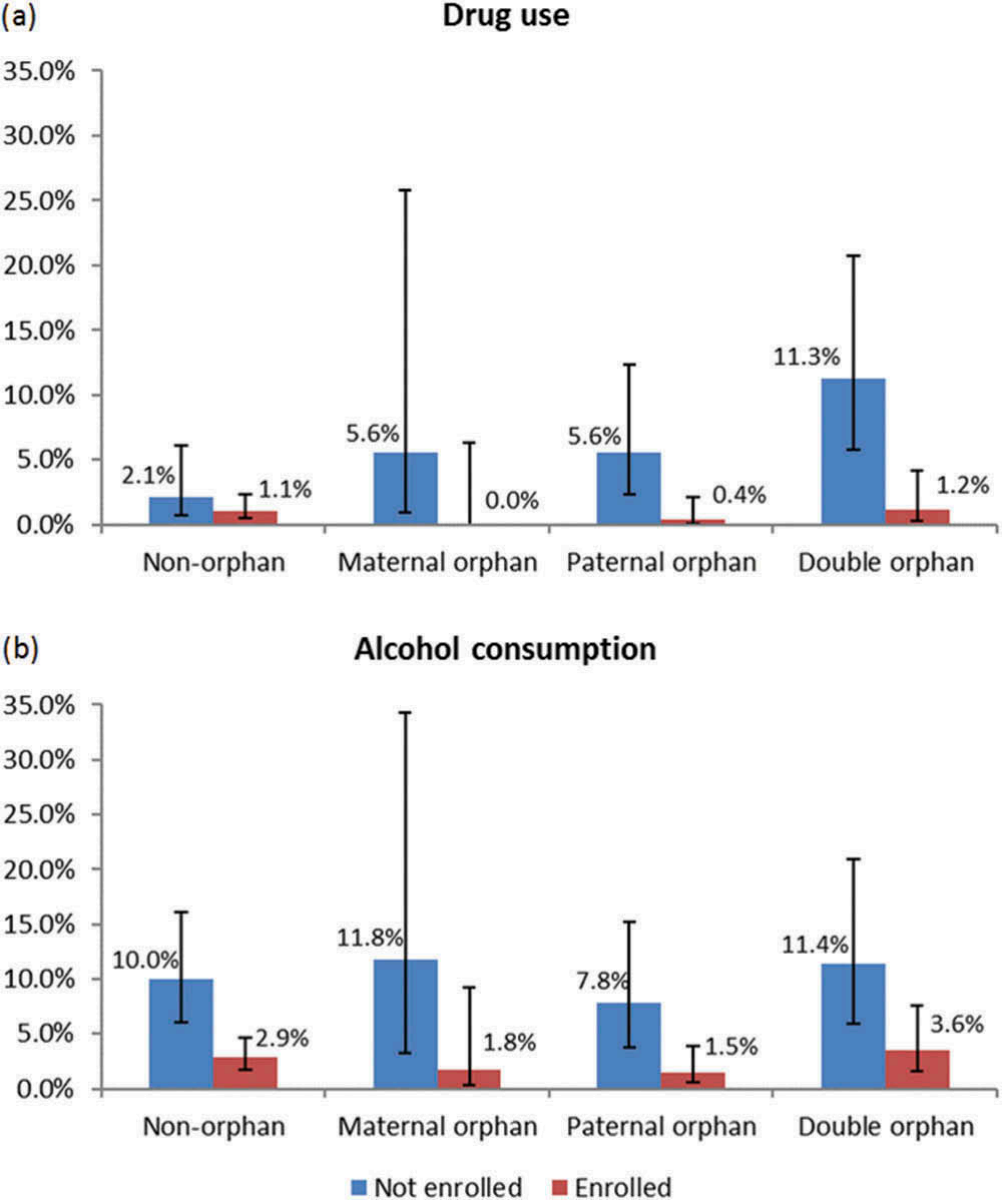
Drug use (a) and alcohol consumption (b) in male orphans and non-orphans under age 19 by school enrolment status.

Finally, we found that school enrolment was significantly associated with lower reporting of taking drugs for pleasure, drinking alcohol, and any risk behaviour in males, before (Table 1) and after (Table 2) adjusting for socio-demographic factors. Additionally, school enrolment was associated with significantly lower levels of substance use for male orphans overall (AOR = 0.32, 95% CI, 0.14–0.75) and for each individual form of orphanhood (Table 2). Similar patterns of effects were found for drugs taken for pleasure and for alcohol (Figure 2) but not for smoking cigarettes. Additionally, school enrolment was negatively associated with ever having had sex, although the significance of this association decreased (*p* < 0.001 vs. *p* = 0.02) after adjusting for substance use (Table 3).

## Discussion

The aim of this paper was to characterise substance use among adolescents from rural Zimbabwe and to investigate whether reductions in substance use might be one pathway through which school enrolment reduces HIV risk in vulnerable and orphaned children. We found that males were significantly more likely to partake in all forms of substance use than females. In both males and females, smoking was the least common form of substance use (0.9% and 0.1%), followed by drug use (3.2% and 0.4%), and finally alcohol consumption (6.4% and 0.7%). A 2010 study of 15-year-old male and female Zimbabweans found the same trends in substance use, albeit with higher levels of smoking (14.5% and 5.6%), drug (15.4% and 11.1%), and alcohol (18.1% and 14.5%) use than those reported here (Peltzer, 2010). The higher levels of substance use in the 2010 study may be due to the inclusion of the large cities of Harare and Bulawayo.

When ascertaining whether orphans were at an increased risk for substance use compared with non-orphans, we found that males who were maternal or double orphans were significantly more likely to have taken drugs for pleasure than non-orphans. Similar results have been found in South Africa, where males who were paternal or double orphans were more likely to have consumed alcohol, and females who were paternal orphans were more likely to have taken drugs (Meghdadpour et al., 2012), but, to our knowledge, this is the first study to document an enhanced risk of drug use among orphans in Zimbabwe. The potential for increased vulnerability of orphans to risky behaviour, including substance use, has been documented before (UNICEF, 2005), as parental death often leaves youth with fewer physical, financial, and emotional resources (Foster & Williamson, 2000; Kembo, 2010; Meghdadpour et al., 2012). Stress, anxiety, feelings of isolations, lack of support, and the absence of role models, all of which may occur with the loss of a parent (Nyamukapa et al., 2010), influence risk behaviours in adolescents (Cluver, Gardner, & Operario, 2007; Perrino, González-Soldevilla, Pantin, & Szapocznik, 2000).

These risk behaviours of substance use can potentially lead to increased HIV risk behaviours, such as those (sexual debut, early sexual debut, number of non-regular partners, and engaging in transactional sex) we saw here. Strong associations between substance use and sexual risk behaviours have been reported previously and include smoking, drinking, and drug use being associated with sexual risk behaviours in general (Duncan, Strycker, & Duncan, 1999; Jackson, Sweeting, & Haw, 2012; Tu, Lou, Gao, Li, & Zabin, 2012), early sexual debut (Lowry et al., 1994), and number of sexual partners (Lowry et al., 1994; Shrier, Emans, Woods, & DuRant, 1997). Binge drinking has been associated with a higher number of partners (Guo et al., 2002), while more generally alcohol consumption has been associated with sexual debut, multiple sexual partners, and unprotected sex, all of which lead to an increased risk of HIV infection (Ayisi et al., 2000; Bassett et al., 1996; Clift et al., 2003; Lewis et al., 2005; Mnyika, Klepp, Kvale, & Ole-King’Ori, 1997; Myer, Mathews, & Little, 2002; Somsé, Chapko, & Hawkins, 1993; Zachariah et al., 2003). The increased risk of HIV infection may be attributable to the social consequences of substance use, which facilitates unprotected sex. The physiological effects of substance use on decision-making (Dingle & Oei, 1997; Simons, Maisto, & Wray, 2010; Steele & Josephs, 1990), the altered expectations of condom use when using substances (Gálvez-Buccollini et al., 2008; Maisto, Carey, Carey, & Gordon, 2002), and certain personality types being more likely to engage in all forms of risky behaviours (Cooper, Wood, Orcutt, & Albino, 2003; Hagger-Johnson, Bewick, Conner, O’Connor, & Shickle, 2011; Newcomb, Clerkin, & Mustanski, 2011), may increase the likelihood of risky sex and therefore HIV infection. Substance use is also likely to be on the causal pathway between orphanhood and increased risk of HIV acquisition as the significant association of orphanhood with higher numbers of partners disappeared after adjusting for substance use, implying that substance use may be a mediating factor for orphans.

To verify the plausibility of education offsetting the occurrence and effects of substance use, we investigated the relationship between education and substance use, and education and sexual risk behaviours. Among males, we found that school enrolment was negatively associated with taking drugs, drinking alcohol, and a combined measure that represented any form of substance use. These associations held true when investigated in only those males who had been orphaned, suggesting that school enrolment is a positive force for vulnerable adolescents as well as adolescents overall. Previous studies in the United States (Reininger et al., 2005) and in South Africa (Flisher, Parry, Evans, Muller, & Lombard, 2003; Meghdadpour et al., 2012) have also shown school-related factors to be protective against substance use. School attendance could decrease substance use through connectedness in the form of bonds with teachers and peers (Flisher et al., 2003; Operario, Cluver, Rees, MacPhail, & Pettifor, 2008). In addition, schooling may increase the chances of finding employment, and may therefore reduce risky behaviour. Not being enroled in school, which we found here to be associated with all forms of substance use bar smoking, may also alter how and where peer relationships are formed, with friendships coming from higher risk venues as opposed to from school (Meghdadpour et al., 2012). School enrolment was also associated with decreased likelihood of sexual debut, but this effect weakened (*p* < 0.001 vs. *p* = 0.02) when substance use was adjusted for, providing evidence that substance abuse is on the causal pathway between education and sexual risk behaviour. Given the associations between education and substance use, and substance use and sexual risk behaviours, and the mediating effects of substance use, a possible pathway through which education could reduce HIV risk in young adults is suggested.

A limitation of the study is the use of cross-sectional data, which may have led to the associations found in the data being due to reverse causation. Although previous work has examined the effect of substance use on educational attainment (Grant et al., 2012; King, Meehan, Trim, & Chassin, 2006; McGue, Iacono, Legrand, Malone, & Elkins, 2001), a review of the literature on the pathways to substance use in adolescents in Canada highlighted academic underachievement and poor attendance as two factors leading to substance use in teenagers (Paglia & Room, 1999), supporting the logic for causation we used herein. Similarly, previous work has also shown that substance use is typically initiated before sexual debut occurs (Tu et al., 2012). The study also relied on self-reported measures, which may have been subject to social desirability bias caused by a potential under-reporting of both substance use and sexual risk behaviours. Additionally, the study was limited by the low prevalence of certain risk behaviours, particularly among women, making it not possible to accurately assess associations in these groups.

## Conclusions

This study elucidates a potential mechanism through which schooling can decrease risky sexual behaviours: by reducing alcohol and drug use which has in turn been tied to risky sexual behaviours. Future work should therefore investigate the role of education and schools as a protective factor not only against HIV infection but also against substance abuse. As trials of cash transfer programmes have shown, the true power of structural interventions lies in how health and behaviour can be affected by addressing the social, political, or economic context of a situation (Baird, Garfein, McIntosh, & Özler, 2012; Ranganathan & Lagarde, 2012; Robertson et al., 2013).

## Supplementary Material

Supplementary_Table_1.doc
