# Supplementary material for: Education, substance use, and HIV risk among orphaned adolescents in Eastern Zimbabwe
Source: Vulnerable Child Youth Stud. 2017 Oct 2;12(4):360–74. doi: 10.1080/17450128.2017.1332398 (PMC5679749; doi:10.1080/17450128.2017.1332398)
Supplement: Supplementary_Table_1.doc [file RVCH_A_1332398_SM0035.doc]

Table S1. Characteristics of young adults included in study

|  | Males (n=1609)  Mean (IQR), % (95% CI) |  | Females (n=1665)  Mean (IQR), % (95% CI) |
| --- | --- | --- | --- |
| Mean age | 16.8 (16-18) |  | 16.7 (16-18) |
|  |  |  |  |
| Community type |  |  |  |
| Town | 15.2% (13.5-17.0) |  | 15.7% (14.0-17.5) |
| Commercial estate | 20.9% (18.9-22.9) |  | 20.8% (18.9-22.8) |
| Subsistence farming | 41.1% (38.7-43.5) |  | 41.3% (40.0-43.7) |
| Roadside trading centre | 22.7% (20.7-24.8) |  | 22.1% (20.1-24.1) |
|  |  |  |  |
| Religion |  |  |  |
| Christian | 54.2% (51.7-56.6) |  | 54.3% (51.9-56.7) |
| Traditional | 0.2% (0.0-0.5) |  | 0.2% (0.0-0.4) |
| Spiritual | 25.6% (23.5-27.8) |  | 29.8% (27.6-32.0) |
| Other | 14.4% (12.7-16.1) |  | 14.7% (13.0-16.4) |
| None | 5.5% (4.4-6.7) |  | 1.0% (0.5-1.5) |
|  |  |  |  |
| Orphanhood |  |  |  |
| Non-orphan | 51.0% (48.5-53.5) |  | 51.3% (48.9-53.7) |
| Maternal | 5.3% (4.2-6.4) |  | 6.4% (5.2-7.6) |
| Paternal | 26.2% (24.1-28.4) |  | 26.6% (24.5-28.8) |
| Double | 17.5% (15.7-19.4) |  | 15.7% (13.9-17.4) |
|  |  |  |  |
| Enrolled in school^†^ | 76.7% (74.5-79.0) |  | 65.5% (63.0-67.9) |
|  |  |  |  |
| Substance use |  |  |  |
| Smoking | 0.9% (0.5-1.4) |  | 0.1% (0.0-0.3) |
| Drug use | 3.2% (2.3-4.0) |  | 0.4% (0.0-0.6) |
| Alcohol consumption | 6.4% (5.2-7.6) |  | 0.7% (0.3-1.1) |
|  |  |  |  |
| Sexually active | 6.4% (5.2-7.6) |  | 24.4% (22.3-26.5) |
|  |  |  |  |
| HIV risk behaviours |  |  |  |
| Sexual debut before age 15 | 0.5% (0.2-0.8) |  | 2.8% (1.5-2.9) |
| Condom use at last sexual encounter^‡^ | 70.9% (62.0-79.7) |  | 9.7% (6.8-12.5) |
| Engaging in transactional sex^‡^ | 4.9% (0.7-9.0) |  | 2.5% (1.0-4.0) |
| Mean number of non-regular partners in the last 30 days^‡^ | 2.0 (1-2) |  | 0.2 (0-0) |

^†^Ages 15-18

^‡^Among those who have had sexual debut
